# Supplementary figures and images for: Inter- and intraspecific variation in the Artibeus species complex demonstrates size and shape partitioning among species
Source: PeerJ. 2021 Jul 12;9:e11777. doi: 10.7717/peerj.11777 (PMC8280882; doi:10.7717/peerj.11777)

**A**

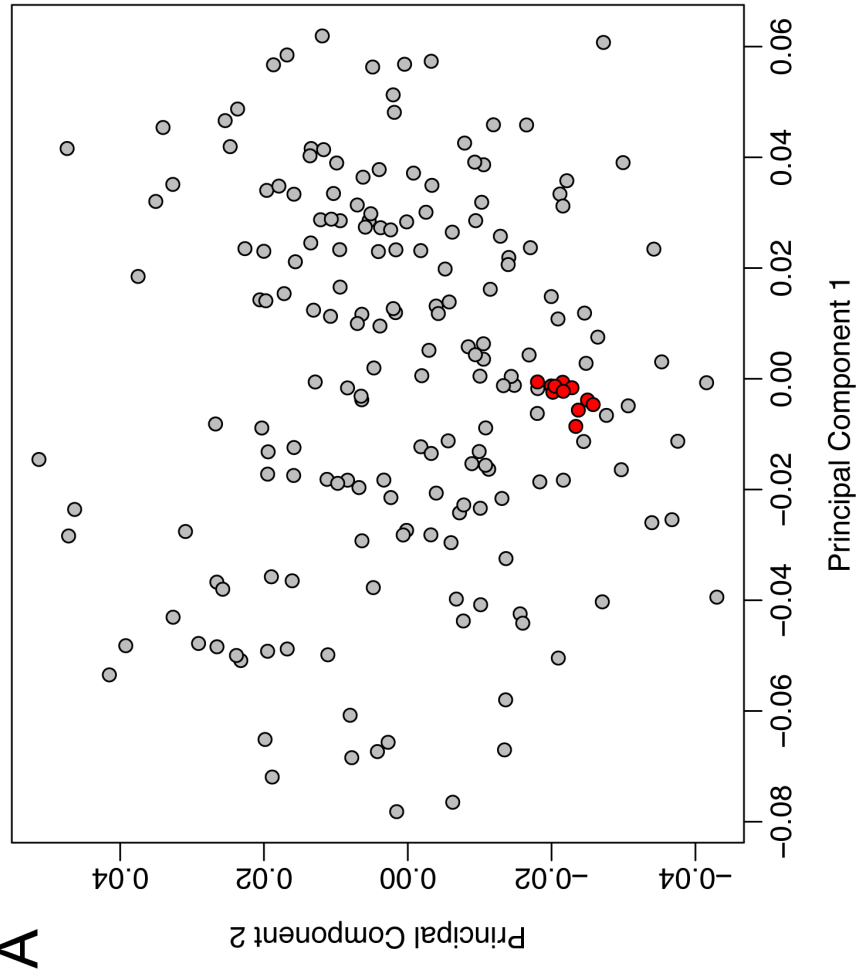

**B**

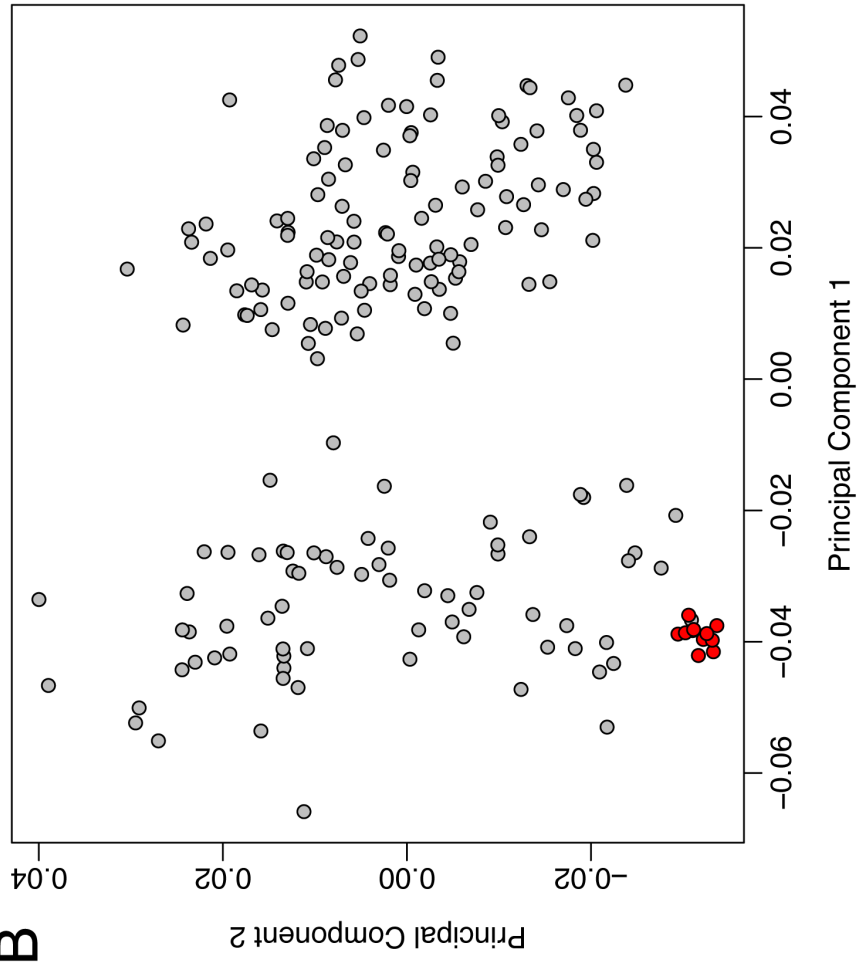

Supplement: Supplemental Information 1 — To assess error in the data, one specimen was selected at random and landmarked 10 times. That specimen (red) was then plotted in PC morphospace with all other specimens (gray) demonstrating minimal within individual landmarking error compared to between individual differences. [file peerj-09-11777-s001.pdf]

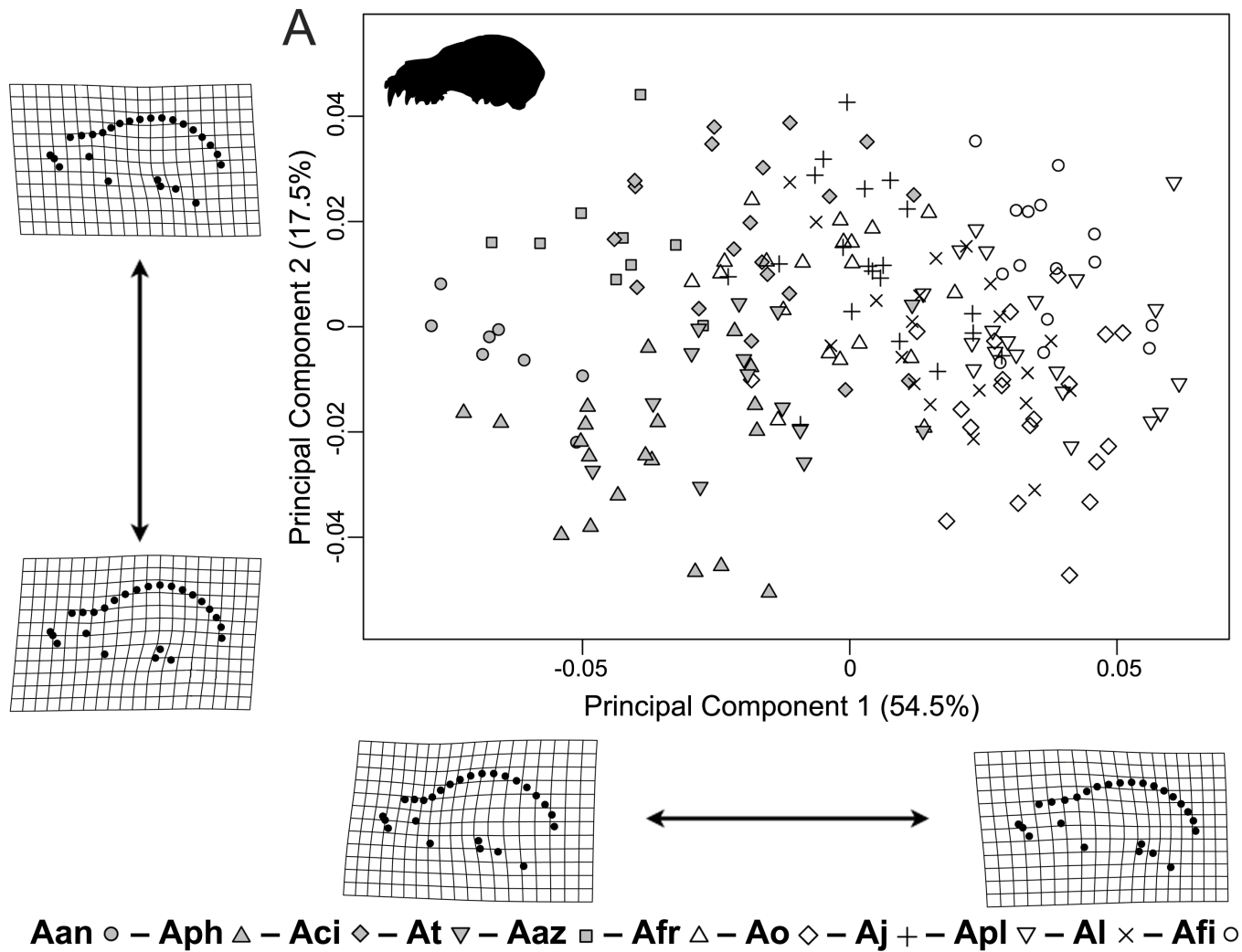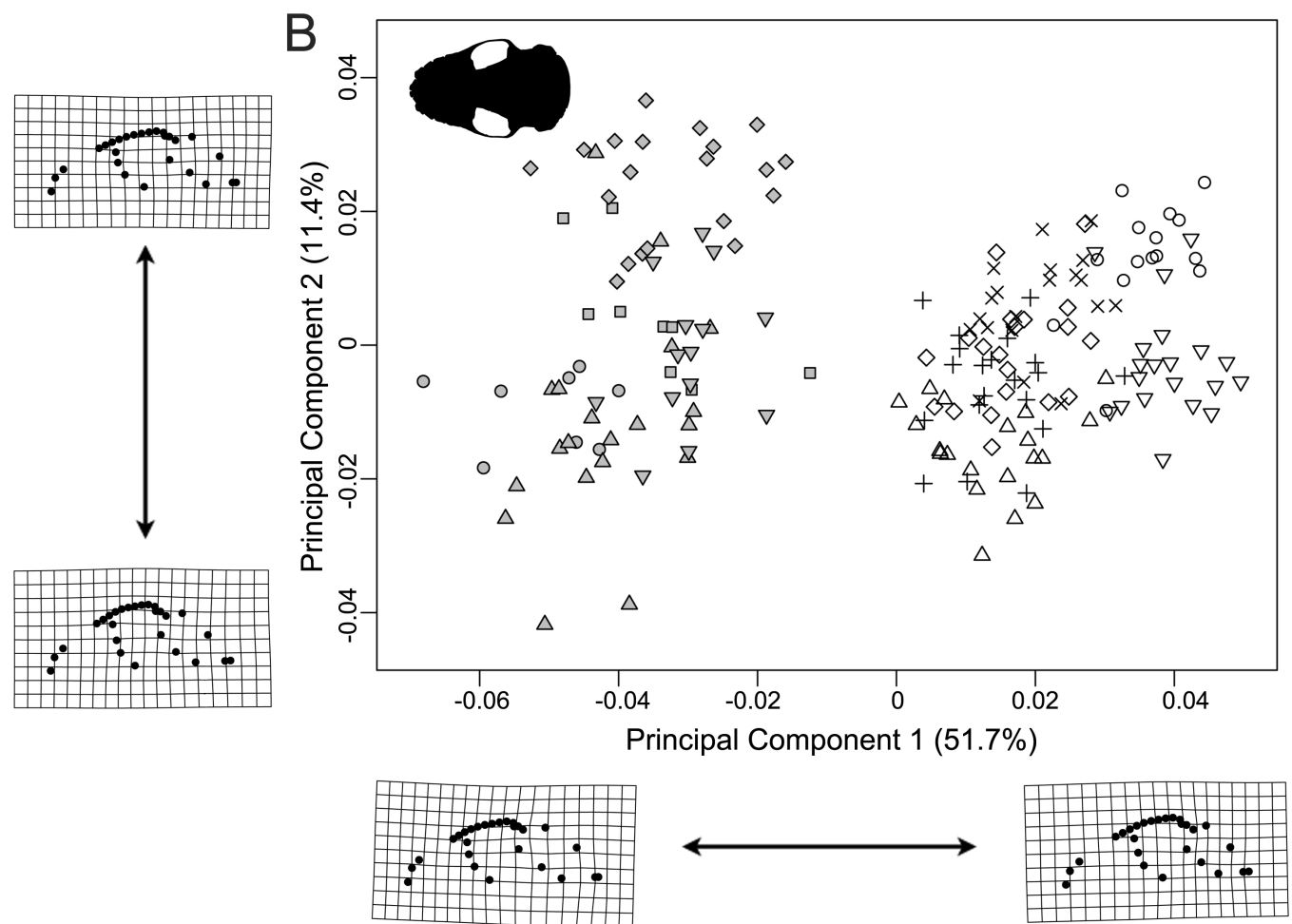

Supplement: Supplemental Information 2 — Thin-plate spline grids represent shape change along principal component 1 and 2. Shapes and species codes refer to individual taxa within the Artibeus species complex. Species codes refer to Fig. 1 and shapes are shown next to species codes within the figure. [file peerj-09-11777-s002.pdf]

Aan Aaz Aci Afi Afr Aj Al Ao Apl At

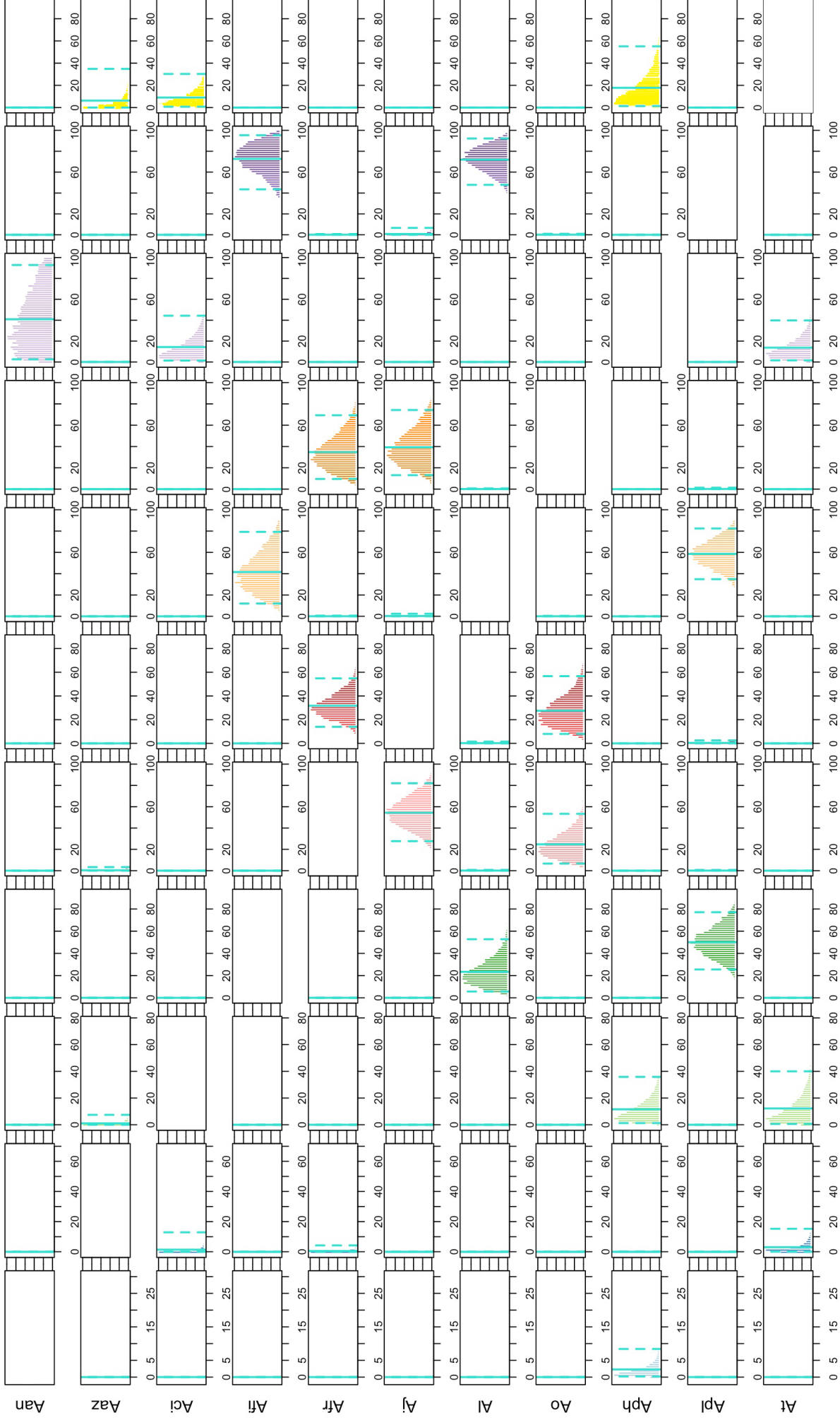

Overlap Probability (%) -- Niche Region Size: 95%

Supplement: Supplemental Information 3 — X-axis shows the probability (1–100) of niche overlap of the species in the row versus the species in the column. The solid vertical turquoise line represents the mean probability and the hashed vertical turquoise lines represent the 95% confidence intervals. For species pairs where the mean is 0, there is no overlap between species. Aan = Artibeus anderseni, Aaz = A. aztecus, Aci = A. cinereus, Afi =A. fimbriatus, Afr = A. fraterculus, Aj = A. jamaicensis, Al = A. lituratus, Ao = A. obscurus, Aph = A. phaeotis, Apl = A. planirostris, At = A. toltecus. [file peerj-09-11777-s003.pdf]
