# Supplementary material for: Inter- and intraspecific variation in the Artibeus species complex demonstrates size and shape partitioning among species
Source: PeerJ. 2021 Jul 12;9:e11777. doi: 10.7717/peerj.11777 (PMC8280882; doi:10.7717/peerj.11777)
Supplement: Supplemental Information 4 — X-axis shows the probability (1–100) of niche overlap of the species in the row versus the species in the column. The solid vertical turquoise line represents the mean probability and the hashed vertical turquoise lines represent the 95% confidence intervals. For species pairs where the mean is 0, there is no overlap between species. Aan = Artibeus anderseni, Aaz = A. aztecus, Aci = A. cinereus, Afi = A. fimbriatus, Afr = A. fraterculus, Aj = A. jamaicensis, Al = A. lituratus, Ao = A. obscurus, Aph = A. phaeotis, Apl = A. planirostris, At = A. toltecus. [file peerj-09-11777-s004.pdf]

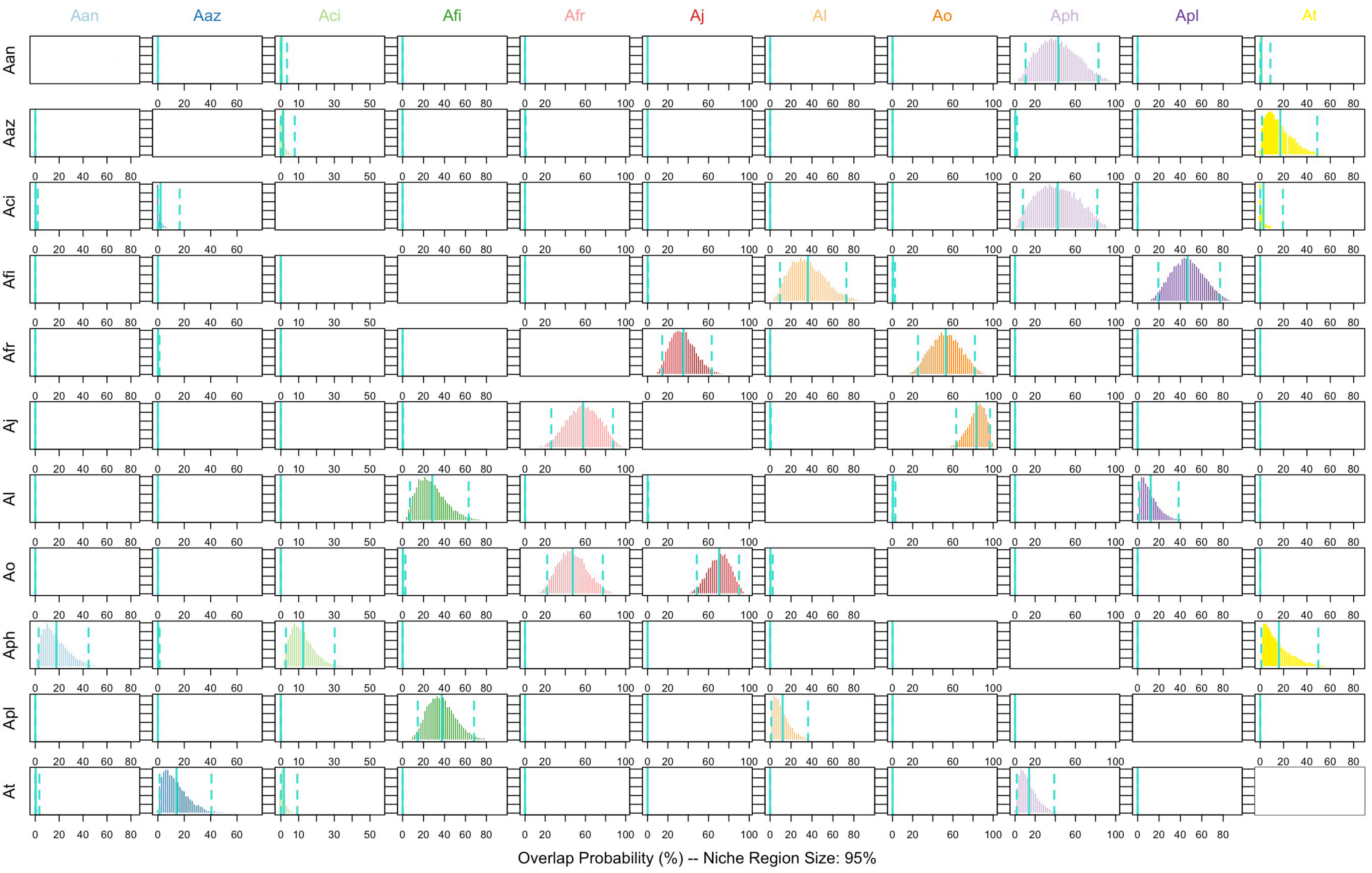

Aan
Aaz
Aci
Afi
Afr
Aj
Al
Ao
Aph
Apl
At
Overlap Probability (%) -- Niche Region Size: 95%
