# Supplementary material for: Inter- and intraspecific variation in the Artibeus species complex demonstrates size and shape partitioning among species
Source: PeerJ. 2021 Jul 12;9:e11777. doi: 10.7717/peerj.11777 (PMC8280882; doi:10.7717/peerj.11777)
Supplement: Supplemental Information 5 — Principal component morphospaces (PC1 x PC2) for species means in (A) lateral and (B) ventral views with the species phylogeny from Rojas, Warsi & Dávalos (2016) plotted on top of points. White circles represent nodes on the phylogeny. Colors correspond to species colors in figure 1 of the main text. Disparity through time analyses for (C) lateral and (D) ventral views. Solid line represents observed data while dotted line represents Brownian motion. Both views show an increase in disparity at 5 mya at the split between the small and large Artibeus. [file peerj-09-11777-s005.pdf]

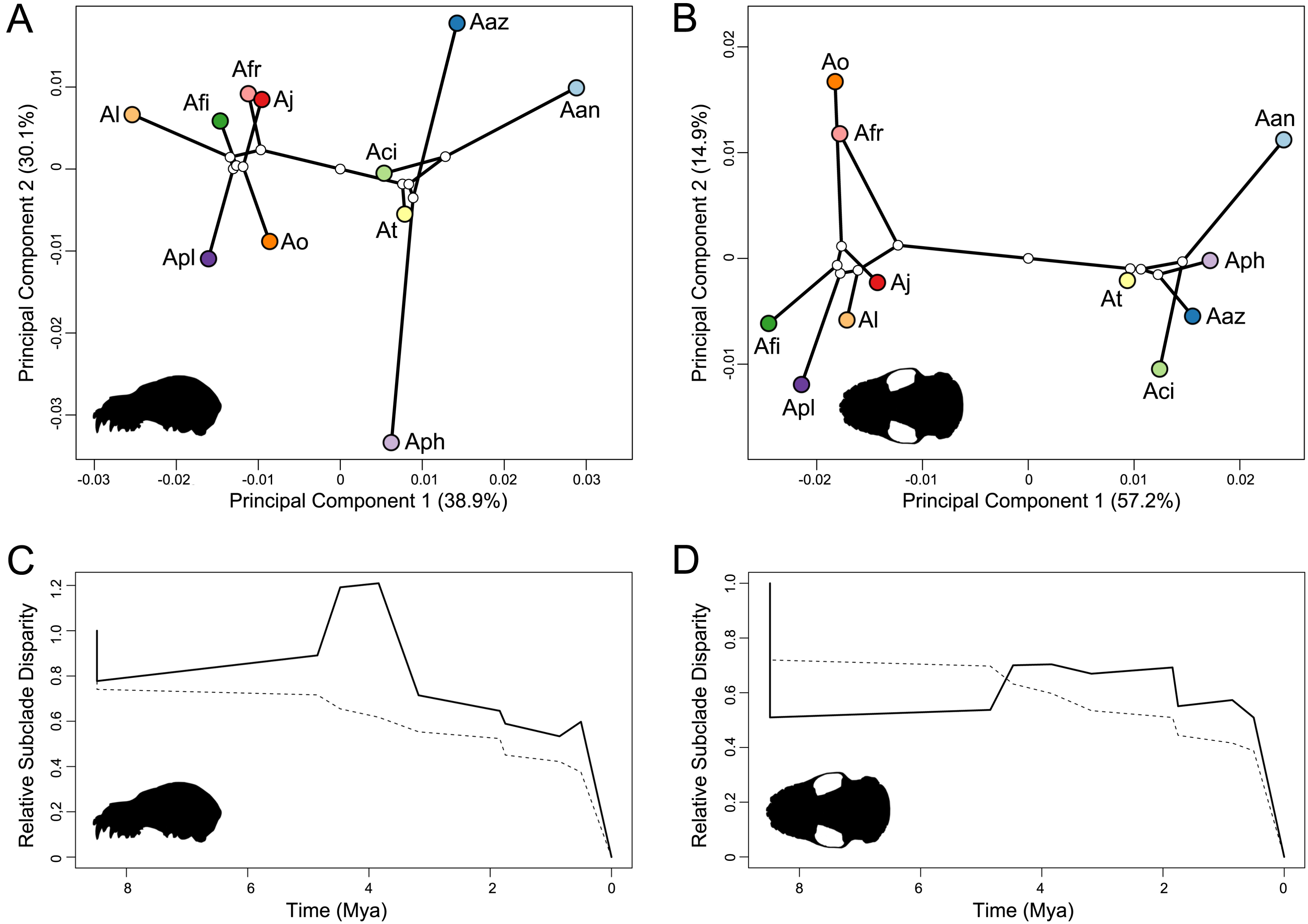
A
B
C
D
Principal Component 1 (38.9%)
Principal Component 2 (30.1%)
Principal Component 1 (57.2%)
Principal Component 2 (14.9%)
Relative Subclade Disparity
Time (Mya)
Aaz
Aan
Afr
Aj
Afi
Al
Aci
At
Apl
Ao
Aph
Afi
Apl
Aci
-0.03
-0.02
-0.01
0
0.01
0.02
0.03
1.2
1.0
0.8
0.6
0.4
0.2
8
6
4
2
