# Supplementary material for: Inter- and intraspecific variation in the Artibeus species complex demonstrates size and shape partitioning among species
Source: PeerJ. 2021 Jul 12;9:e11777. doi: 10.7717/peerj.11777 (PMC8280882; doi:10.7717/peerj.11777)
Supplement: Supplemental Information 11 [file peerj-09-11777-s011.pdf]

Los murciélagos neotropicales de hoja nasal (filostómidos) son una de las familias mamíferas más diversas y *Artibeus* spp. es el género filostómido con más especies. A pesar de su riqueza de especies, trabajos anteriores sobre el cráneo de *Artibeus* usando morfometría lineal, han descubierto variación interespecífica limitada. Esta falta de variación de forma sugiere la ausencia de partición de nicho en todas las especies, muchas de las cuales con frecuencia se encuentran en simpatría. Usando métodos geométricos morfométricos bidimensionales en una muestra grande de cráneos de once especies del complejo de especies *Artibeus* (*Artibeus* y *Dermanura*), el estudio actual demuestra una variación craneal interespecífica, dimorfismo sexual en tamaño y forma y variación geográfica intraespecífica. La mayoría de las especies mostraron un tamaño y forma únicos, lo cual sugiere que cada especie aprovecha recursos ecológicos ligeramente distintos. Asimismo, el dimorfismo sexual en tamaño y forma fue significativo en el complejo de especies *Artibeus*. Los *Artibeus* machos y hembras tienen estrategias de forrajeo específicos al sexo, ya que los machos comen cerca de sus perchas y las hembras comen lejos de sus perchas. La presencia de dimorfismo sexual craneal en el complejo de especies *Artibeus*, al igual que estudios anteriores que muestran que diferentes tamaños y durezas de fruta están relacionados con las diferentes formas craneales de filostómidos, indican que los machos y las hembras pueden estar utilizando recursos de alimento diferentes, llevando a morfotipos craneales divergentes. Mas estudios ayudaran a confirmar esta nueva hipótesis. Por último, se encontró una considerable variación de tamaño geográfica en una muestra grande intraespecífica de cráneos de *Artibeus lituratus*. No obstante, esta variación no estuvo relacionada con la latitud y en cambio puede estar ligada a los factores ambientales locales. Trabajo adicional sobre la ecología y comportamiento del complejo de especies *Artibeus* subyacentes a la variación morfológica descubierta en este estudio hará posible un mejor entendimiento acerca de cómo el grupo ha logrado su diversidad actual.
